# Highly diverse *nirK* genes comprise two major clades that harbour ammonium-producing denitrifiers

**DOI:** 10.1186/s12864-016-2465-0

**Published:** 2016-02-29

**Authors:** Decleyre Helen, Heylen Kim, Bjorn Tytgat, Willems Anne

**Affiliations:** Laboratory of Microbiology (LM-UGent), Department of Biochemistry and Microbiology, Ghent University, K.L. Ledeganckstraat 35, B-9000 Ghent, Belgium

**Keywords:** Classes of NirK, cNirK, CuNIR, Sequence diversity, Genomic linkage

## Abstract

**Background:**

Copper dependent nitrite reductase, NirK, catalyses the key step in denitrification, i.e. nitrite reduction to nitric oxide. Distinct structural NirK classes and phylogenetic clades of NirK-type denitrifiers have previously been observed based on a limited set of NirK sequences, however, their environmental distribution or ecological strategies are currently unknown. In addition, environmental *nirK*-type denitrifiers are currently underestimated in PCR-dependent surveys due to primer coverage limitations that can be attributed to their broad taxonomic diversity and enormous *nirK* sequence divergence. Therefore, we revisited reported analyses on partial NirK sequences using a taxonomically diverse, full-length NirK sequence dataset.

**Results:**

Division of NirK sequences into two phylogenetically distinct clades was confirmed, with Clade I mainly comprising *Alphaproteobacteria* (plus some *Gamma*- and *Betaproteobacteria*) and Clade II harbouring more diverse taxonomic groups like *Archaea*, *Bacteroidetes*, *Chloroflexi*, *Gemmatimonadetes*, *Nitrospirae*, *Firmicutes*, *Actinobacteria*, *Planctomycetes* and *Proteobacteria* (mainly *Beta* and *Gamma*). Failure of currently available primer sets to target diverse NirK-type denitrifiers in environmental surveys could be attributed to mismatches over the whole length of the primer binding regions including the 3′ site, with Clade II sequences containing higher sequence divergence than Clade I sequences. Simultaneous presence of both the denitrification and DNRA pathway could be observed in 67 % of all NirK-type denitrifiers.

**Conclusion:**

The previously reported division of NirK into two distinct phylogenetic clades was confirmed using a taxonomically diverse set of full-length NirK sequences. Enormous sequence divergence of *nirK* gene sequences, probably due to variable *nirK* evolutionary trajectories, will remain an issue for covering diverse NirK-type denitrifiers in amplicon-based environmental surveys. The potential of a single organism to partition nitrate to either denitrification or dissimilatory nitrate reduction to ammonium appeared to be more widespread than originally anticipated as more than half of all NirK-type denitrifiers were shown to contain both pathways in their genome.

**Electronic supplementary material:**

The online version of this article (doi:10.1186/s12864-016-2465-0) contains supplementary material, which is available to authorized users.

## Background

Canonical denitrification is the conversion of fixed nitrogen to a gaseous end product with concomitant energy conservation [1, 2]. It is a facultative respiratory pathway in which nitrate, nitrite, nitric oxide and nitrous oxide are sequentially reduced to dinitrogen gas, each step catalysed by one or more metallo-enzymes [2]. This important process has been intensely studied, both experimentally and in situ, as it is part of the global nitrogen cycle and contributes to the loss of fixed nitrogen from the environment as well as the emission or mitigation of nitrous oxide, i.e. a greenhouse gas that has a 310x greater global warming potential than carbon dioxide and is involved in ozone destruction [3]. Although denitrification has long been considered as a modular process [2], with certain denitrifiers lacking either nitrate reductase and/or nitrous oxide reductase, whole genome sequences revealed the existence of extremely truncated versions of the pathway with only one enzyme or discontinuous chains of two or more enzymes [4, 5]. This raises the semantic though relevant question of when an organism should be considered as denitrifier. We propose that, adhering to the original description of the process, a denitrifier *sensu stricto* should be capable of acquiring energy from at least the one-electron reduction of nitrite to the gaseous nitric oxide, meaning it should at least contain a nitrite reductase.

This key enzyme in the denitrification process exists in two evolutionary unrelated variants, *i.e*. copper containing nitrite reductase NirK and cytochrome *cd*_*1*_ nitrite reductase NirS encoded by the *nirK* and *nirS* gene respectively [2, 6]. Some organisms contain more than one *nirK* or *nirS* gene copy [7] and, recently, both types of Nir were found to not be mutually exclusive [4], although functionality of the two different nitrite reductase types within one organisms still needs confirmation. NirS-type denitrifiers are often assumed to be predominant in the environment, while NirK-type denitrifiers cover more diverse taxa [2]. It should, however, be noted that (i) very few studies have attempted to quantify both NirK and NirS-type denitrifiers in environmental surveys, with most only including *nirS* [8–10], and (ii) *nirK* gene sequence divergence exceeds that of *nirS* genes resulting in constrained applicability of the currently available *nirK* primers, leading to an underestimation of NirK-type denitrifiers [11]. Both types of denitrifiers are assumed to respond differentially to environmental gradients with NirK-type denitrifiers exhibiting greater habitat selectivity [12–15]. Co-occurrence patterns of *nirS*, *nor* and *nos* genes suggested shared regulatory mechanisms that may constrain loss of *nor* and *nos* in *nirS*-type denitrifiers [4]. In contrast, no such genomic linkage patterns were observed for NirK indicating that NirK-type denitrifiers are more likely to perform incomplete denitrification. Taken together with previously reported positive correlations between NirK abundance and nitrous oxide emissions [16] and the negative relationship between the ratio nitrous oxide/dinitrogen gas and NirS-type denitrifiers [17], NirK-type denitrifiers might contribute significantly more to nitrous oxide emission than their NirS counterparts.

The large divergence in NirK sequences from taxonomically distant as well as closely related NirK-type denitrifiers has not yet been well-characterized despite the need for accurate assessment of abundances and community structure of these potential nitrous oxide emitters. While there are conserved catalytic domains shared by all NirK proteins, substantial variation exists in the primary structure between different members of the NirK protein family. Copper-containing nitrite reductase NirK (also sometimes designated as cNirK/CuNIR) is a periplasmic, homotrimeric enzyme with each monomer typically containing two copper centres, T1Cu ligated by four amino acid residues (two His, Cys and Met) and T2Cu ligated by three His and a water molecule [18]. The enzyme receives one electron at the T1Cu site from an electron donor and catalyses the one-electron reduction of nitrite to nitric oxide at the T2Cu site. Moreover, an essential hydrogen bond network including Asp and His around T2Cu functions as proton donor to the substrate [19]. Three different classes of NirK-type nitrite reductases have been designated previously based on their structure. (i) Soluble periplasmic Class I NirK found in some *Alphaproteobacteria* can be divided into two subclasses based on their optical absorption spectrum and sequence identity: green Nir types (described for *Achromobacter cycloclastes* and *Alcaligenes faecalis* S6) and blue Nir types (described for *Alcaligenes xylosoxidans*) [20]. (ii) Outer-membrane bound Class II NirK was described for *Neisseria gonorrhoeae* [21]. (iii) More divergent NirK sequences found in some Gram-positive bacteria and the ammonia oxidizing bacterium *Nitrosomonas europaea* [22]. Furthermore, the NirK of *Hyphomicrobium denitrificans* with an additional cupredoxin domain at the N-terminus [20, 23] and those of *Burkholderia*, *Ralstonia* and *Bdellovibrio* with a C-terminus extension containing a class I *c*-type heme domain [20], do not fit the current classification. In addition to structural differences, phylogeny has been used to delineate two NirK clades supported by distinct amino acid motifs, *i.e*. TRPHL and SSFHV/I/P, around the active site His residue [24]. Clade I was found to harbour Class I NirK sequences, while Clade II NirK contained more taxonomically diverse NirK-type denitrifiers including some belonging to Class II NirK.

It is clear that an unambiguous NirK classification is lacking. Furthermore, currently available primers only target Class I *nirK* sequences [11, 24]. So, although NirK-type denitrifiers are potential nitrous oxide emitters, the predominance of various NirK classes or clades in the environment cannot be unequivocally and systematically evaluated. This is unfortunate as it could unveil different ecological strategies or environmental distributions among denitrifiers harbouring distinct clades or classes of NirK, as was recently demonstrated for the two distinct clades of nitrous oxide reductase NosZ [25–27]. Therefore, we revisited and extended the NirK analyses of Jones and colleagues [24], by performing detailed sequence and phylogenetic analyses on NirK sequences from fifteen different phyla in light of the structurally different NirK classes described so far. The previously proposed grouping of NirK in two distinct phylogenetic clades was confirmed and further underpinned by Clade II specific indels. The potential to partition nitrate between denitrification and dissimilatory nitrate reduction to ammonium appeared common to denitrifiers from both clades. Furthermore, evolutionary trajectories underpinning the extremely high NirK sequence divergence and consequences for *nirK* primer coverage were considered.

## Results and discussion

### Proposed NirK classification in two clades based on phylogenetic and sequence analysis

**Fig. 1:**
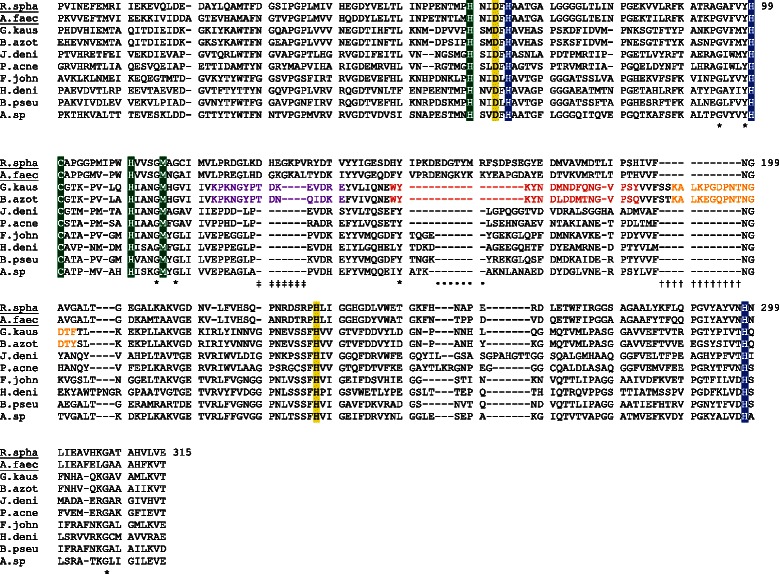
Trimmed multiple sequence alignment of NirK from *Rhodobacter sphaeroides* ATCC 17025 (R. spha), *Alcaligenes faecalis* S-6 (A.faec), G*eobacillus. kaustophilus* HTA426 (G.kaus), *Bacillus azotoformans* LMG 9581 T (B.azot), *Jonesia denitrificans* DSM 20603 (J.deni), *Propionibacter acnes* C1 (P.acne), *Flavobacteria johnsoniae* UW101 (F.john), *Halomonas denitrificans* ATCC 35960 (H.deni), *Burkholderia pseudomallei* 668 (B.pseu) and *Azospirillum* sp. B506 (A.sp). Sequences belonging to Clade I NirK are underlined. Amino acid numbering was based on the full-length NirK sequence of *Rhodobacter sphaeroides* ATCC 17025. The linker, tower and extra loop specific for *Bacillus* sp. are given in purple, red and orange respectively. Copper binding motives T1Cu and T2Cu are indicated in green and blue respectively, active site residues Asp and His required for nitrite reducing activity in yellow, conserved regions are indicated by *, deletions specific to Clade II NirK sequences are indicated by ‡ and • and the *Bacillus* specific insertion by †

Copper dependent nitrite reductase NirK encoded by the *nirK* gene catalyses the key step of the denitrification pathway, *i.e*. the reduction of nitrite to the gas nitric oxide. In environmental surveys, this gene has been used as a proxy for denitrification despite previously reported limitations of conventional *nirK* primers [11, 28] resulting in the underestimation of both the presence and abundance of NirK-type denitrifiers in different environments. Broad taxonomic diversity of NirK-type denitrifiers as well as the impressive divergence among NirK sequences both account for the limited coverage of current primers. A NirK dataset composed of 267 full-length NirK sequences, extracted from whole genomes, from fifteen different Bacterial and Archaeal phyla was used for phylogenetic and sequence analysis. Prior to use of the dataset, all NirK sequences were verified for the presence of core Cu-binding sites (His59, His64, His99, Cys100, His110, Met115 and His298) and the active site residues Asp62 and His237 required for nitrite reducing activity (Fig. 1) [19, 29]. Comparison of NirK sequences with other copper oxidases lacking Nir activity (laccases and ascorbate oxidases) indicated that the residues Asp62, His64, Cys100, His110, Met115 and His237 were not conserved. Based on this observation, we were convinced that all NirK sequences included in this study were indeed nitrite reductases. NirK sequence lengths varied substantially from 304 to 995 aa, with the majority of NirK sequences ranging between 300 and 560 aa, and some Actinobacterial sequences around 700–990 aa.

**Fig. 2:**
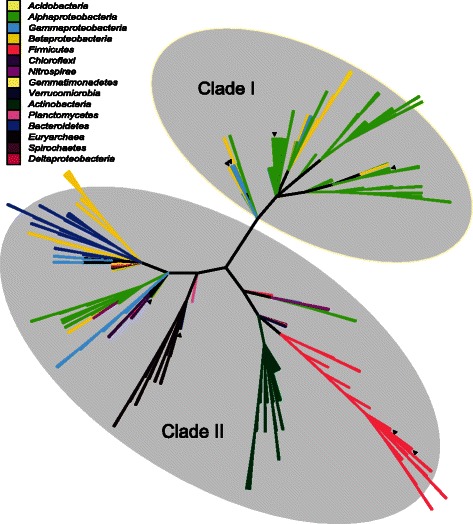
Unrooted bifurcating neighbour joining tree of full-length NirK sequences obtained from whole genomes. NirK Clade I encompassed *Alphaproteobacteria* and some *Gamma*- and *Betaproteobacteria*, while NirK Clade II comprised *Firmicutes*, *Actinobacteria*, *Euryarchaea*, *Chloroflexi*, *Bacteroidetes*, *Nitrospirae*, *Gemmatimonadetes*, *Planctomyces*, *Verrucomicrobia* and *Proteobacteria*. The same bifurcating tree in cladogram format with an estimation of reliability based on bootstrap analysis can be found in supplementary information (Additional file 1). Physiologically characterized denitrifying strains are indicated by ▼: *Bacillus azotoformans* LMG 9581, *Bacillus bataviensis* LMG 21833 [39], *Haloferax denitrificans* ATCC 35960 [40], *Hyphomicrobium denitrificans* ATCC 51888 [77], *Shewanella loihica* PV-4 [44], *Shewanella denitrificans* OS217 [78], *Rhodobacter sphaeroides* ATCC 17025 [79], *Alcaligenes faecalis* S-6 [80]. The grey ovals represent the two previously proposed clades of NirK sequences

**Fig. 3:**
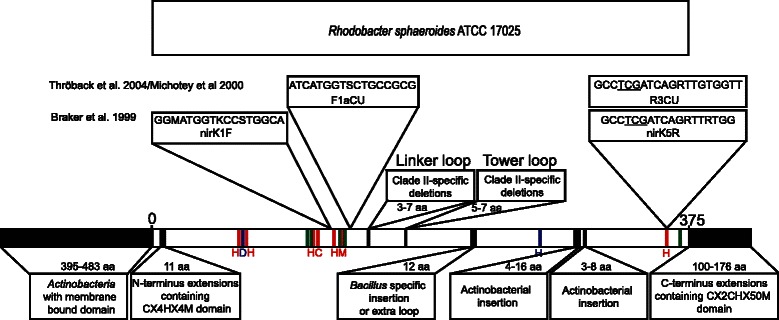
Schematic illustration of sequence length variation, primer target site of two frequently used primer sets F1aCu-R3Cu [33] and nirK1F-nirK5R [34], and different types of indels and extensions observed in 267 NirK sequences. The 375 aa long sequence of *Rhodobacter sphaeroides* ATCC 17025 (pdb accession number 1ZV2) was used as reference. Size and, if possible, information on taxon or clade defined by these indels or extensions are represented. Copper binding sites, essential active site residues and conserved regions are indicated in red, blue and green respectively. The copper binding His included in the reverse primer target sites is underlined. Abbreviations of copper binding and active site residues are presented due to limited space, with H: His, C: Cys, D:Asp and M:Met

Neighbour joining phylogenetic analysis resulted in the distinction of two NirK clades (Fig. 2): (i) Clade I mainly comprised of *Alphaproteobacteria* (plus some *Gamma*- and *Betaproteobacteria*) and (ii) Clade II harboured more diverse taxonomic groups like *Archaea*, *Bacteroidetes*, *Chloroflexi*, *Gemmatimonadetes*, *Nitrospirae*, *Firmicutes*, *Actinobacteria*, *Planctomycetes* and *Proteobacteria* (mainly *Beta* and *Gamma*). A maximum likelihood analysis was also performed and resulted in a similar grouping (data not shown). Using an expanded dataset of over 250 full-length NirK sequences, we thus confirm the previously described existence (based on 147 partial *nirK* sequences) of two distinct NirK clades [24], and propose the systematic inclusion of both NirK Clades I and II in further environmental surveys. Clade I corresponded to the previously described Class I NirK nitrite reductases [20, 24], while NirK sequences previously defined as belonging to Class II or unclassified, all clustered together in Clade II. Interestingly, this grouping into two clades is underpinned by small indels specific to Clade II (Fig. 3), specifically two deletion regions (7aa) which coincided with the linker and tower loop previously observed in *Bacillus* and *Neisseria* species (Figs. 2 and 3) [21, 23, 30]. The extra loop regions unique to NirK from *Bacillaceae* (Figs. 1 and 3) [30] were observed in all *Bacillus* sp. with exception of the three *Paenibacillus* species. *Actinobacteria* also contained additional insertions although the size, location and presence/absence of these insertions were variable (Fig. 3 and Additional file 1). We also found that the TRPHL and SSFHV/I/P motifs around the active site His237 (Fig. 2, Additional file 1) thought to be unique to Clade I and II respectively [24] could not be used as good distinguishing features, as the corresponding regions within each clade were much more diverse than previously described. Approximately 61 % of all Clade I NirK contained a TRPHL motif, with other previously undescribed motifs being SRPHL (32 %), SYPHL (3 %), SRIHL (1 %) and TR/YPHI (3 %). More than half of all Clade II NirK (61 %) contained a SSFHV/I/P motif while 39 % comprised other and more diverse motifs such as CH/TFHV (4 %), LSFHV/I (5 %), SNFHV/I/P (5 %), SSFHL (5 %), with especially Actinobacterial and Archaeal NirK containing previously undescribed motifs (Additional file 1). As could be expected from their interaction to form a hydrogen bound network involved in proton supply for substrate reduction [21], substantial variation in AA motifs around the other essential T2Cu centre residue, Asp 62 (Fig. 2), were also observed (Additional file 1). Distinction between NirK Clade I and II based on signal peptide, as previously observed for the two clades of NosZ [26], was not found.

Strikingly, the overall mean NirK similarity within the dataset was extremely low (only 10 %), with especially Clade II being defined by low sequence similarity (16 % compared to 66 % in Clade I). This even higher than previously appreciated level of NirK sequence divergence and above mentioned substantial sequence size variation may be partly attributed to the high incidence of N- and C-terminus extensions in approximately 30 % of all NirK sequences. C-terminus extensions comprising a class I *c-*type heme domain CX_2_CHX_50_M previously only reported in NirKs from *Burkholderia*, *Ralstonia* and *Bdellovibrio* [20], were observed in 39 other NirK sequences derived from *Spirochaetes*, *Verrucomicrobia*, *Alpha*-, *Beta*-, *Delta*- and *Gammaproteobacteria* (Fig. 3 and Additional file 1). Recent NirK analysis of the commensal bacterium *Neisseria weaveri* revealed high sequence similarity between the C-terminus *c*-type heme and other Neisserial NirK cytochrome electron carriers, *i.e*. Ccop and *c*_*5*_ cytochromes [31], suggesting it might function as an alternative electron transport route to NirK providing an adaptive advantage in nitrite limiting environments. In contrast, these C-terminus extensions are not found in pathogenic *Neisseria* sp., further underlining their potential adaptive benefit linked to distinct lifestyles. The approximately 900 aa long NirK of *Propionibacterium acnes* was previously found to contain a N-terminus extension comprising a 400 aa predicted transmembrane domain of unknown function and an additional cupredoxin domain, *i.e*. CX_4_HX_4_M (Fig. 3). All Actinobacterial NirK included in this study, were observed to contain the cupredoxin domain, however, not all contained a transmembrane domain (Additional file 1), resulting in substantial sequence length variation within the *Actinobacteria* (900 aa vs. 500 aa). The cupredoxin containing N-terminus extensions were also previously reported in non-actinobacterial *Hyphomicrobium denitrificans* ATCC 51888, *Herpetosiphon aurantiacus* DSM 785 and *Nitrosospira multiformis* ATCC 25196 [20], and were additionally observed here in six other NirK sequences belonging to *Nitrospirae*, *Alpha*- and *Betaproteobacteria* (Additional file 1). The function of the N-terminus cupredoxin domain remains unknown as it located too far away from the catalytic core for effective electron transfer [23] and, moreover, higher catalytic activity was observed in N-terminus T1Cu mutant compared to the wild type [32]. Nevertheless, its conservation and wide occurrence does suggest some adaptive benefits despite its presence being not required for NirK functioning. NirK sequences were not found to contain both N- or C-terminus extensions, which corroborates previous observations [20].

### High sequence divergence causes *nirK* primer coverage issues

**Fig. 4:**
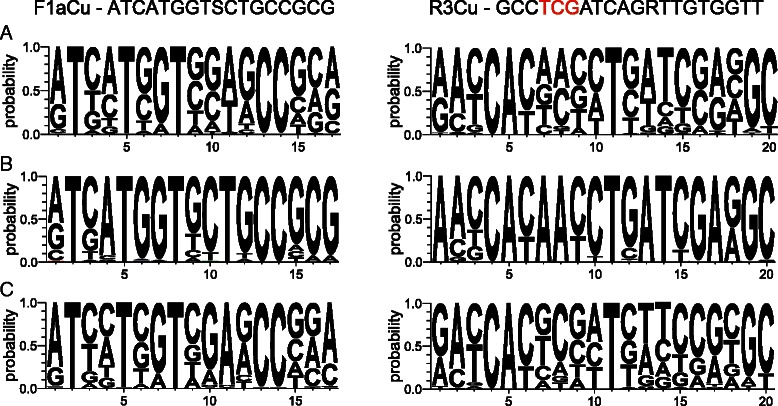
Sequence logo diagrams (5′–3′) depicting the degree of *nirK* sequence variability of F1aCu-R3Cu primer binding regions [33] in (**a**) Clade I + II *nirK*, (**b**) Clade I *nirK*, and (**c**) Clade II *nirK*. The primer sequence is given at the top and copper binding His included in the reverse primers is indicated in red. Diagrams for the primers nirK1F-nirK5R [34] can be found in Additional file 2

Although shotgun sequencing is becoming increasingly affordable for environmental monitoring of ecosystem functions, amplification-based gene sequencing and quantification is still widely performed. Primer coverage is a well-known but continuing problem for successful amplification and sequencing of any gene from environmental or mixed samples and especially for the very diverse *nirK* gene. The most frequently used primers F1aCu-R3Cu [33] and NirK1F-NirK5R [34] bind to supposedly conserved copper binding regions outside the Clade II indels and/or variable Asp or His motifs described higher and shown in Fig. 3, yet still they only detect Clade I sequences [11, 24, 28]. Large sequence divergence seemed to exist throughout the whole *nirK* gene, even at supposedly conservative sites used for primer design (Fig. 4 and Additional file 2). We directly related failure of current primers as broad range tools to mismatches over the whole primer binding regions including the 3′ site. As expected from sequence variation, this was more explicit for Clade II than Clade I sequences (Fig. 4 and Additional file 2).

As the sequence divergence within Clade II was very high and modification of available primers to increase coverage is not straightforward [11], we attempted to design novel *Bacteroidetes* specific *nirK* primers using the current dataset. *Bacteroidetes* NirK sequence divergence averaged around 28 %, making this taxon a better test case for primer design compared to *Actinobacteria* and *Archaea* (sequence divergence of 42 % and 37 % respectively). Although Firmicutes sequence divergence was found to be the lowest (22 %), the *Bacteroidetes* group was chosen as a test group because recently *Geobacillus* specific *nirK* primers [35] were published. The novel primers (Additional file 3) scored significantly better for successful amplification in pure cultures than the traditional ones but unfortunately did not render amplicons from various environmental samples (Additional file 4). Although it cannot be excluded that *nirK*-containing *Bacteroidetes* were only present under the detection limit in these samples, it seems more likely that, despite their high degeneracy, the novel primers were too specific to capture all diversity present (Additional file 3). A similar observation was made for primers targeting denitrifying *Geobacillus* ([35], unpublished data). Wei and colleagues, however, recently succeeded in designing multiple new *nirK* primer sets targeting more diverse Clade I sequences and numerous subgroups within Clade II [36], though the dataset used for primer design was limited to 97 *nirK* sequences and did not contain *Firmicutes. In silico* evaluation of these new primers on the *Bacteroidetes* dataset indicated that the reverse primer did not find a match for all *Bacteroidetes* sequences within the alignment. It is clear that primer coverage, albeit essential for proper experimental design of environmental surveys, remains an issue difficult to resolve. Given the large sequence diversity, universal primers will probably remain elusive. Nevertheless, new attempts to improve *nirK* primer coverage are still needed as quantitative PCR currently represents the only available quantification technique allowing absolute quantification of functional genes in environmental surveys as (shotgun) sequencing techniques are only semi-quantitative. Therefore, combining algorithm-based primer design with continuously updating of functional gene datasets represents the most promising approach for designing different sets of subclade specific *nirK* primers that can be used in combination to optimise coverage.

### Evolutionary processes resulting in high sequence divergence

Phylogenetic analyses of NirK sequences (Fig. 2) showed clustering of NirK-type denitrifiers according to their taxonomic origin for *Alphaproteobacteria* in Clade I, and *Actinobacteria*, *Firmicutes* and *Archaea* within Clade II. Nevertheless, high NirK sequence divergence between closely related NirK-type denitrifiers is common and generally known [5, 24, 37], with Beta- and Gammaproteobacterial clusters also found in Clade I and *Verrucomicrobia*, *Spirochaetes*, *Chloroflexi*, *Beta*-, *Delta*- and *Gammaproteobacteria* sequences spread throughout the entire Clade II (Fig. 2). Limited taxonomic coherence was observed in the NirK phylogeny, which corroborates with previous reports on incongruence between phylogenies of 16S rRNA gene sequences and denitrification genes [22, 24]. However, some taxonomic coherence could be observed for Archaeal, Actinobacterial and Firmicutes NirK sequences, although even in these phyla there were exceptions. For example, *Sulfobacillus acidophilus* TPY did not cluster together with other Firmicutes NirK sequences and was furthermore found to share only 5 % sequence similarity with the other Firmicutes NirK sequences (Additional file 1). Also, the NirK from Gammaproteobacterial *Cellvibrio gilvus* ATCC 13127 grouped within the *Actinobacteria* cluster while that of the Bacteroidetes *Rhodothermus marinus* SG0.5JP17-172 was found in the cluster of the *Archaea*. Jones and colleagues [24] already elaborately discussed that the evolutionary process responsible for these incongruences might not be as simple as horizontal gene transfer. They proposed that gene duplication and subsequent sequence divergence of the different copies and/or gene loss as well as lineage sorting could be primarily responsible. In that light, it is interesting to note that approximately 5 % of all NirK-type denitrifiers in our dataset were found to contain more than one *nirK* gene copy in their genome. Although our observations are biased because of considerable differences in number of genomes available per phylum (Table 1), *Alphaproteobacteria* were mostly found to contain multiple copies, but also some representatives of *Betaproteobacteria*, *Firmicutes*, *Nitrospirae* and even *Archaea*. The majority of these taxa harboured two copies, with exception of *Afipia* sp. 1NLS2 which was previously described to contain three [5]. As was found for *Afipia*, the Betaproteobacterium *Pusillimonas* sp. T7-7 and the Alphaproteobacterium *Ochrobactrum anthropi* ATCC 49188 each harbored two NirK sequences belonging to different clades. All other taxa contained NirK copies belonging to either Clade I or II, with various degrees of divergence (Additional file 1). Clearly, multiple *nirK* copies may provide adaptive advantages to the denitrifiers in changing environments, provided that both genes are expressed as functional nitrite reductases under different physicochemical conditions [7]. Differential loss and unequal evolutionary pressures on both copies could explain the observed incongruent organisms and denitrification gene phylogenies.

**Table 1:** Modularity of denitrification and DNRA observed in the 249 NirK containing genomes covering fifteen different phyla. Percentage complete denitrification (Nir + Nor + Nos), incomplete denitrification (-Nor, −Nos or -Nor/-Nos), DNRA (Nar + NrfA, Nap + NrfA, NrfA, Nar + NirB, Nap + NirB or only NirB), and combined occurrence of both processes (NirK + NrfA and/or NirB) are represented for each phylum separately and over all 249 NirK genomes

	No.of genomes	No. of *nirK* copies per genome	% complete denitrification	% incomplete denitrification			% DNRA						% denitrification and DNRA
		(% of phylum)	*nir* + *nor* + *nos*	-*nor*	-*nos*	-*nor*/-*nos*	*nap* + *nrfA*	*nar* + *nrfA*	*nrfA*	*nap* + *nirB*	*nar* + *nirB*	*nirB*	*nirK* + *nrfA*and/or *nirB*
*Acidobacteria*	1	1 (100)	100	0	0	0	0	0	0	0	0	0	0
*Actinobacteria*	28	1 (100)	0	0	50	50	3.6	7.1	17.9	3.6	39.3	0	64.3
*Alphaproteobacteria*	82	1 (92.7); 2 (6.1) or 3 (1.2)	41.5	1.2	39.0	18.3	15.7	0	0	65.9	11	6.1	78.0
*Bacteroidetes*	21	1 (100)	42.9	19.0	28.6	9.5	9.5	0	42.9	9.5	0	9.5	66.7
*Betaproteobacteria*	39	1 (92.3) or 2 (7.7)	61.5	0	30.8	7.7	2.6	2.6	0	61.5	61.5	5.1	74.4
*Verrucomicrobia*	2	1 (100)	0	50	0	50	100	0	0	50	0	0	100
*Chloroflexi*	5	1 (100)	0	20	0	80	0	0	0	0	0	40	40
*Deltaproteobacteria*	2	1 (100)	0	0	50	50	0	0	50	0	0	50	50
*Euryarcheaota*	13	1 (84.6) or 2 (15.4)	46.2	0	38.5	15.3	0	0	0	0	0	7.7	7.7
*Firmicutes*	27	1 (96.3) or 2 (3.7)	40.8	7.4	25.9	25.9	11.1	11.1	0	37	37	3.7	55.6
*Gammaproteobacteria*	23	1 (100)	19.0	0	33.3	47.7	30.4	4.3	0	47.8	17.4	0	73.9
*Gemmatimonadetes*	1	1 (100)	0	100	0	0	0	0	0	0	0	0	0
*Nitrospirae*	2	1 (50) or 2 (50)	0	0	50	50	0	0	0	0	0	0	0
*Planctomycetes*	1	1 (100)	0	0	0	100	100	100	0	100	100	0	100
*Spirochaetae*	2	1 (100)	50.0	0	0	50.0	0	0	0	50	0	50	100
Total	249	5.3	36.0	4.0	34.8	25.2	12.1	3.2	6.1	42.2	23.7	6.0	66.7

Compared with NirK, it can be assumed that the overall sequence divergence of NirS might be lower as NirS classes based on structural diversity, phylogenetic groups or amino acid motifs have not been described. We hypothesize that the different evolutionary trajectories of both nitrite reductases might potentially be related to the differential spread of the encoding genes over prokaryotic life, with NirS being limited to mostly *Proteobacteria* [24, 36]. However, an additional important feature to consider might be the difference in operon size between both *nir* types. NirS maturation requires the expression of a multiple gene operon consisting of at least three or four *nir* genes [22], while NirK maturation does not require other genes, although sometimes it is accompanied by a second gene encoding the protein NirV [38]. NirV was detected in approximately 61 % of the genomes included in our dataset, mainly in *Proteobacteria* (*Alpha* and *Beta*) and *Bacteroidetes*, confirming that the presence of NirV is not mandatory for NirK maturation [22]. The Firmicutes *Bacillus azotoformans* LMG 9581 [39] and the archaeon *Haloferax denitrificans* ATCC 35960 [40] have both been physiologically shown to perform denitrification, however, no NirV could be detected. Alternatively, it is also possible that NirV was missed in these organisms due to the limited number and diversity of reference sequences available (only *Alphaproteobacteria*). Therefore, we hypothesize that evolutionary pressure on *nirS* is much higher than on *nirK,* as mutations are less likely to be retained by natural selection because of incompatibility of the expressed enzyme with accessory proteins. Differential evolutionary pressures on both *nir* genes might not only explain differential mutation rates but also the higher probability for mobility and gene duplication of *nirK*. Furthermore, compared to *nirK*, occurrence of the *nirS* gene appears to be more often linked to other denitrification genes [4]. Indeed, NirK can be involved in other nitrogen processes such as anaerobic ammonium oxidation (Anammox) [41] and nitrifier denitrification [42], as well as have a function other than anaerobic respiration of nitrogen oxides [43]. So, distinct taxonomic breadth, operon structure and metabolic versatility of *nirS-*and *nirK*-type denitrifiers might contribute to distinct evolutionary trajectories resulting in the higher level of sequence divergence for NirK.

### DNRA is common in both Clade I and II NirK-type denitrifiers

Denitrification and dissimilatory nitrate reduction to ammonium (DNRA), a process also contributing to nitrous oxide emission, were long believed to be performed by distinct microbial populations. However, genome analysis of *Shewanella loihica* [44], *Bacillus azotoformans* [45] and other bacteria [26] have recently revealed the gene inventory for both nitrate reducing processes to be present in one organism. Functional capacity to carry out both nitrate reducing processes and their determining environmental drivers, mainly carbon-to-nitrogen ratio and nitrite concentrations, have since been demonstrated for *S. loihica* [46, 47]. Interestingly, all organisms containing both processes were NirK-type denitrifiers. In our dataset, approximately 67 % of NirK-type denitrifiers were found to harbour both pathways in their genome (Table 1), with 25.9 % belonging to Clade I and 40.8 % to Clade II. Unequal distribution over different phyla was observed for *nrfA* and *nirB*, the genetic markers for respiratory and fermentative DNRA respectively and the genes coding for the periplasmic pentaheme cytochrome *c* and the cytoplasmic NADH-dependent nitrite reductases respectively (Table 1). However, inadequate representation of certain phyla due to a lower number of genomes may have obscured a clear picture of their occurrence. The metabolic versatility rendered from the ability to use both processes is a clear competitive advantage in variable environments, especially those with changing carbon and/or nitrogen loads [46, 48, 49]. Looking at both cytoplasmic and periplasmic nitrate reductase genes (*narG* and *napA* respectively) (Table 1), a weak but significantly negatively correlated co-occurrence was found between *narG* and *nrfA* (R = −0.23, *p* < 0.05) which agrees with the general assumption that, if a nitrate reductase is present, nitrate reduction in DNRA organisms proceeds typically via the periplasmic nitrate reductase Nap (Table 1) [50]. This also nicely fits with the similar preference of Nap and NrfA for low nitrate concentrations [48, 51]. The differential preference of Nar and NrfA for high and low nitrate concentrations respectively also agrees with this negative co-occurrence pattern [48, 51]. The inventory of denitrification genes in all 249 NirK containing genomes varied enormously (Table 1) and recently reported observations such as the simultaneous presence of NirK and NirS, the high occurrence of only Nir and Nor in *Actinobacteria* and the observation of highly truncated versions of the denitrification pathway [4] were confirmed here. Our analyses therefore suggest that NirK denitrifiers are not only more likely to contribute more to nitrous oxide emissions due to the higher occurrence of truncated denitrification [4] but also because of their ability to carry out DNRA. Hitherto, the mechanism of nitrous oxide production by DNRA bacteria remains unknown, however, some evidence indicates that nitrous oxide production might mainly result from nitrite detoxification during DNRA activity. For example, it was demonstrated for both *E. coli* and *Salmonella enterica* serovar *Typhimurium* that the nitrite conversion to nitric oxide occurred only after nitrate was depleted, required molybdate—the essential cofactor of NarGHI nitrate reductase -, continued in *nirB*^−^ mutants [52] but was lacking in *narG*^−^ mutants [53, 54]. Later mutagenesis experiments in *E. coli* could, however, not confirm the involvement of NarGHI in nitric oxide production [55]. Instead, NirB and NrfA appeared to be involved in the production of nitric oxide [55] and their relative importance appeared to be dependent on the nitrite concentration [56]. Produced nitric oxide might subsequently be converted to nitrous oxide to maintain NO homeostasis through the presence of (i) quinol-dependent nitric oxide reductase qNor which is known to be present in both denitrifiers and non-denitrifiers [45, 57, 58], (ii) NrfA [59], (iii) flavohemoglobin hmp [60] or (iv) the cytoplasmic flavorubredoxin NorV and its associated oxidoreductase NorW [61]. Future mutagenic studies are nevertheless required to further elucidate and comprehend the different nitrous oxide producing pathways during DNRA activity.

## Conclusions

This study confirms the existence of the two phylogenetic distinct clades of NirK based on a taxonomically diverse set of full-length NirK sequences, with Clade I harbouring Class I NirK and, Clade II containing a more diverse set of structurally different NirK. We propose a systematic usage of Clade I and Clade II designation in NirK sequence analyses of environmental surveys to ascertain potential ecophysiological differences between NirK-type denitrifiers from each clade. In amplicon-based surveys, enormous *nirK* sequence divergence, due to variable *nirK* evolutionary trajectories, will remain the major cause of limited primer coverage. The simultaneous presence of both the denitrification and DNRA pathway appears to be more widespread than originally anticipated, as more than half of all NirK-type denitrifiers were shown to contain both pathways in their genome.

## Methods

### Acquisition, processing and analysis of NirK sequences for database building

In total, a set of 267 NirK sequences, representing 249 microbial genome sequences across 15 phyla, were downloaded from the microbial genome (complete and DRAFT) database of GenBank. A two-step search was performed to acquire full-length NirK sequences as many genes are often misannotated or annotated in different ways. First, protein BLASTs were performed using NirK sequences with known crystal structure (*Alcaligenes faecalis* [62], *Rhodobacter sphaeroides* [63], *Neisseria gonorrhoeae* [21], *Achromobacter xyloxidans* [64], *Geobacillus kaustophilus* [30]), with additionally the NirK sequence of *Bacillus azotoformans* LMG 9581^T^ [45] to increase matches with NirK sequences of Gram-positive bacteria. Then, search terms such as nitrite reductase copper, NirK and copper containing nitrite reductase were used to find additional NirK sequences via the gene search module of the integrated microbial genome (IMG) database [65] with following settings: Filters-Gene product Name (inexact), Sequencing status-All finished, Permanent Draft and Draft, Domain-Bacteria.

### Sequence and phylogenetic analyses

All NirK sequences were aligned using the MUSCLE algorithm (Settings gap penalties: −2.9 Gap Open, 0 Gap extend, 1.2 Hydrophobicity multiplier) [66] and the alignment was manually refined in Mega 6.06 [67]. Sequences were checked for the presence of the copper-binding sites of T1Cu and T2Cu, the active site residues and previously described conserved regions. Redundancy of the dataset was reduced by calculating the pairwise differences between all sequences using the number of differences method in Mega 6.06 [67] and eliminating different strains of the same species with identical NirK sequences. Manual inspection of the NirK alignment was performed to check for N- and/or C-terminus extensions and to identify differences in insertions, deletions and conserved regions between the different taxonomic groups included. The alignment was trimmed to cover the maximum region shared by all NirK sequences before neighbour joining phylogenetic analysis in Mega 6.06 [67], resulting in the exclusion of N- and C-terminus extension from further analysis. Following settings were applied during phylogenetic analysis: Phylogeny test—Bootstrap (1000 replicates), Substitution model—number of differences method based on amino acids and Gaps/Missing data treatment—Complete deletion. In addition, a maximum likelihood analysis was performed using the FastTree tree building software [68] with the Whelan and Goldman evolutionary model, bootstrap testing (1000 replicates) and the discrete gamma model with 20 rate categories.

Signal peptide prediction was done as described by Emanuelsson et al. [69]: SignalP 4.1 was used for prediction of secretory signal proteins [70], TatP 1.0 for twin-arginine translocation signal proteins [71], LipoP 1.0 for lipoprotein signal proteins [72], and SecretomeP 2.0 for non-classical protein secretion [73]. When both Tat and Sec signal peptides were predicted for a single sequence, the one with the highest D-value was retained. Prediction servers did not cover archaeal sequences.

### Co-occurrence patterns of NirK, NrfA, Nar and Nap

To query NirK-encoding genomes to detect the presence of either (in)complete denitrification and/or DNRA, a diverse set of homologous amino acid sequences of functionally characterized NapA, NarG, NirS, NrfA, Nor and NosZ (Additional file 5) was used to build a pBlast database for each gene in CLC genomics Wb 7.5. NirB sequences were directly downloaded from the RAST server [74] and checked for the presence of essential metal binding motifs using *Escherichia coli* K12 (P08201) and *Bacillus subtilis* 162 (P42435) as reference sequences. A NirV database was built to assess the occurrence of NirV in taxonomically diverse NirK-type denitrifiers. A pBlast search was performed using the genome of a NirK-type denitrifier as a query against the different BLAST databases in CLC genomics Wb 7.5 (Expect value of 0.00001, Existence 11- Extensions 1 as Gap cost and BLOSUM62 as matrix). In case of a hit, the obtained functional AA sequence from the genome of a NirK-type denitrifier was also blasted against the NCBI database to obtain information on the domains present which was used as an extra confirmation of its functional gene identity. This way a presence/absence table was constructed containing information on the presence/absence of all functional genes tested in all 249 microbial genomes included in this study. This table served as a starting point for co-occurrence and Pearson correlation analyses that were performed in default settings using the co-occur and Hmisc package in R 3.1.2 [75, 76].

### Availability of supporting data

All the supporting data are included as additional files. The alignment used for the construction of the phylogenetic trees was deposited in Dryad (http://dx.doi.org/10.5061/dryad.n9f8j).

## Additional files

Additional file 1:
**Bifurcating neighbour joining tree of 267 NirK sequences representing 249 genomes.** Bootstrap values < 70 % after performing 1000 replications are not shown. NirK Clade I encompassed *Alphaproteobacteria* and some *Gamma*- and *Betaproteobacteria*, while NirK Clade II comprised of *Firmicutes*, *Actinobacteria*, *Euryarchaea*, *Chloroflexi*, *Bacteroidetes*, *Nitrospirae*, *Gemmatimonadetes*, *Planctomyces*, *Verrucomicrobia* and *Proteobacteria*. Additional information is added to the cladogram in the following order: taxonomic position, signal peptide prediction, observed amino acid motif around the essential T2Cu His and Asp amino acids (previously undescribed His motifs are in bold), the presence of DNRA (based on presence of NrfA and/or NirB), the presence of denitrification (DNF indicates presence of NirS), characteristic insertions or deletions and N- or C-terminus extensions. Specifically for *Actinobacteria*, an * was added if no corresponding transmembrane domain was present in the N-terminus extension. Full terms of abbreviations included are DNRA: dissimilatory nitrate/nitrite reduction to ammonium, DNF: denitrification, NCS: non-classical secretion, Del.: deletion, Ins.: insertion, Und.: undefined, Tat: twin-arginine signal peptide, Sec: secretory signal peptide, Lipo: lipoprotein signal. Physiologically characterized denitrifying strains are indicated by ▼: *Bacillus azotoformans* LMG 9581, *Bacillus bataviensis* LMG 21833 [39], *Haloferax denitrificans* ATCC 35960 [40], *Hyphomicrobium denitrificans* ATCC 51888 [77], *Shewanella loihica* PV-4 [44], *Shewanella denitrificans* OS217 [78], *Rhodobacter sphaeroides* ATCC 17025 [79], *Alcaligenes faecalis* S-6 [80]. (EPS 5314 kb) Additional file 2:
**Sequence logo diagrams (5′–3′) depicting the degree of** ***nirK*** **sequence variability of nirK1F-nirK5R primer binding regions [** 34 **] in (a) Clade I + II** ***nirK*** **, (b) Clade I** ***nirK***, **and (c) Clade II** ***nirK***. The primer sequence is given at the top and copper binding His included in the reverse primers is indicated in red. An indel in the forward primer binding region of Clade II is present. (EPS 1078 kb) Additional file 3:
**Comparison of qualitative performance of traditional and novel** ***nirK*** **primers on** ***Bacteroidetes*** **and non-** ***Bacteroidetes*** **strains.** ^a^ +, PCR product of expected size, −, no PCR amplification, n/d, not determined. Expected size of PCR product is shown in parentheses. Total genomic DNA from a subset of eight strains was extracted according to the guanidium-thiocyanate-EDTA-sarkosyl method (Pitcher et al. 1989) and amplification was performed under following conditions: 25 μl reactions containing 2.5 μl PCR buffer (10x), 2.5 μl dNTPs (2 mM), 2 μl primer (10 μM), 0.5 μl Taq polymerase (1u/μl) and 25 ng DNA extract. Temperature-time profiles were as follows: initial denaturation at 95 °C for 2 min, followed by 35 cycli of 95 °C for 30 s, variable T_a_ depending on primer set for 1 min and 72 °C for 1 min. Final extension was performed at 72 °C for 10 min. ^b^ *nirK* primer pairs previously described by Hallin and Lindgren [33] ^c^ *nirK* primer pairs previously described by Braker et al. [34] ^d^ negative control, organisms known to contain *nirS* ^e^ positive control used in *nirK* gene amplification with traditional primers ^f^ Environmental samples included are soil, estuarine sediments and activated sludge. Identical temperature-time profiles and PCR mixes were used, with exception of the addition of BSA (1.26 μl of 2 mM concentration). (XLS 28 kb) Additional file 4:
***Bacteroidetes*** **specific** ***nirK*** **primers designed in this study and their degree of degeneracy.** ^a^ New *Bacteroidetes* primers designed using the 20 *Bacteroidetes nirK* gene sequences included in this study. *In silico* assessment of the six primer sets (forward primer Bact4Fa or Bact4F with reverse primer Bact4R, Bact3R or Bact1R) on our dataset, using the ‘*Find motif sequence*’ option in Mega 6.06 [67], indicated that the use of Bact4Fa compared to Bact4F as forward primers covered more *Bacteroidetes nirK* sequences. The level of matches with non-*Bacteroidetes nirK* sequences was very low (1.5–3.8 %) for all newly designed primer sets. (XLS 27 kb) Additional file 5:
**Overview of sequences used for Nap, Nar, NirS, Nor, Nos and NirV database building in CLC genomics Wb 7.5 to screen NirK genomes for the presence of these nitrogen cycling genes.** Unique identifiers were included for each sequence and refer to the accession number of the protein database (pdb), UniProtKB/SwissProt or Genbank. NirB sequences were automatically obtained from the RAST server, and are therefore not included in this table. Multiple copies of certain genes of *Bacillus azotoformans* and *Bacillus bataviensis* were included. (XLS 34 kb)
