# Erratum to: Highly diverse nirK genes comprise two major clades that harbour ammonium-producing denitrifiers

**DOI:** 10.1186/s12864-016-2812-1

**Published:** 2016-07-29

**Authors:** Helen Decleyre, Kim Heylen, Bjorn Tytgat, Anne Willems

**Affiliations:** Laboratory of Microbiology (LM-UGent), Department of Biochemistry and Microbiology, Ghent University, K.L. Ledeganckstraat 35, B-9000 Ghent, Belgium

## Erratum

The original article was published in *BMC Genomics* 2016 **17**:155

The online version of the original article can be found under doi:10.1186/s12864-016-2465-0

The online version of the original article can be found at http://dx.doi.org/10.1186/s12864-016-2465-0.

**Erratum to**: *BMC Genomics* 2016, **17**:155 doi: 10.1186/s12864-016-2465-0

Unfortunately in the original publication [1] some of the authors’ first names and surnames were swapped. The correct list of authors is Helen Decleyre, Kim Heylen, Bjorn Tytgat and Anne Willems.
